# Divided attention does not affect the acquisition and consolidation of transitional probabilities

**DOI:** 10.1038/s41598-020-79232-y

**Published:** 2020-12-31

**Authors:** Kata Horváth, Csenge Török, Orsolya Pesthy, Dezso Nemeth, Karolina Janacsek

**Affiliations:** 1 Doctoral School of Psychology, ELTE Eötvös Loránd University, Izabella utca 46, Budapest, 1064 Hungary; 2 Institute of Psychology, ELTE Eötvös Loránd University, Izabella utca 46, Budapest, 1064 Hungary; 3 Brain, Memory and Language Research Group, Institute of Cognitive Neuroscience and Psychology, Research Centre for Natural Sciences, Magyar Tudósok Körútja 2, Budapest, 1117 Hungary; 4 Lyon Neuroscience Research Center, Inserm U1028 - CNRS UMR5292, Université de Lyon, Centre Hospitalier Le Vinatier - Bâtiment 462 - Neurocampus 95 Boulevard Pinel, 69675 Bron Cedex, Lyon, France; 5 Centre for Thinking and Learning, Institute for Lifecourse Development, School of Human Sciences, Faculty of Education, Health and Human Sciences, University of Greenwich, 150 Dreadnought, Park Row, London, SE10 9LS UK

**Keywords:** Neuroscience, Psychology

## Abstract

Statistical learning facilitates the efficient processing and prediction of environmental events and contributes to the acquisition of automatic behaviors. Whereas a minimal level of attention seems to be required for learning to occur, it is still unclear how acquisition and consolidation of statistical knowledge are affected when attention is divided during learning. To test the effect of divided attention on statistical learning and consolidation, ninety-six healthy young adults performed the Alternating Serial Reaction Time task in which they incidentally acquired second-order transitional probabilities. Half of the participants completed the task with a concurrent secondary intentional sequence learning task that was applied to the same stimulus stream. The other half of the participants performed the task without any attention manipulation. Performance was retested after a 12-h post-learning offline period. Half of each group slept during the delay, while the other half had normal daily activity, enabling us to test the effect of delay activity (sleep vs. wake) on the consolidation of statistical knowledge. Divided attention had no effect on statistical learning: The acquisition of second-order transitional probabilities was comparable with and without the secondary task. Consolidation was neither affected by divided attention: Statistical knowledge was similarly retained over the 12-h delay, irrespective of the delay activity. Our findings can contribute to a better understanding of the role of attentional processes in and the robustness of visuomotor statistical learning and consolidation.

## Introduction

Statistical learning refers to the recognition and acquisition of probability-based associations among stimuli^1–5^. This learning process facilitates the efficient processing and prediction of environmental events and contributes to the acquisition of automatic behaviors and skills, such as language, dance, or typing. It can operate on visual or auditory input and with its help, we can process and acquire temporally or spatially distributed associations^5–12^. Statistical learning typically occurs incidentally and the acquired knowledge remains mostly implicit^13–15^. Although it is well-established that statistical learning can occur without intention, i.e., automatically, one of the main challenges in this field is to characterize how statistical learning and attention interact and how previous results concerning these two fundamental cognitive processes could be integrated [see^5,12^]. Whereas a minimal level of attention (to process the relevant stimuli) seems to be required for learning to take place^14,16^, the effect of divided attention on statistical learning is still unclear and understudied^5^. Moreover, it remains unexplored how statistical knowledge acquired under divided attention is consolidated in the post-learning offline period. Therefore, here we aimed to test how divided attention affects statistical learning and the consolidation of the acquired statistical knowledge.

Divided attention is most often investigated by dual-task designs, that is, by testing whether performance is affected in the primary task of interest while participants simultaneously perform a secondary task^17,18^. Previous studies testing the effect of divided attention on statistical learning with dual-task designs led to mixed findings, although the following pattern seems to be emerging overall. When the secondary task is applied to the same stimulus stream and the processing of stimuli is intact (e.g., when participants are instructed to search for a given type of visual shape, while visual statistical learning takes place within the same stream of stimuli), statistical learning appears to be unaffected^14,18, cf. 19,20^. In contrast, when the secondary task operates on different stimuli than the primary task or some stimuli become unattended (e.g. completing a secondary working memory task at the same time as the visual statistical learning task), statistical learning appears to be hindered^16, cf. 19,21–24^. It is important to note, however, that the latter results might reflect the effect of selective instead of divided attention (i.e., when participants have to switch attention between attended and ignored stimuli^17^).

**Figure 1:**
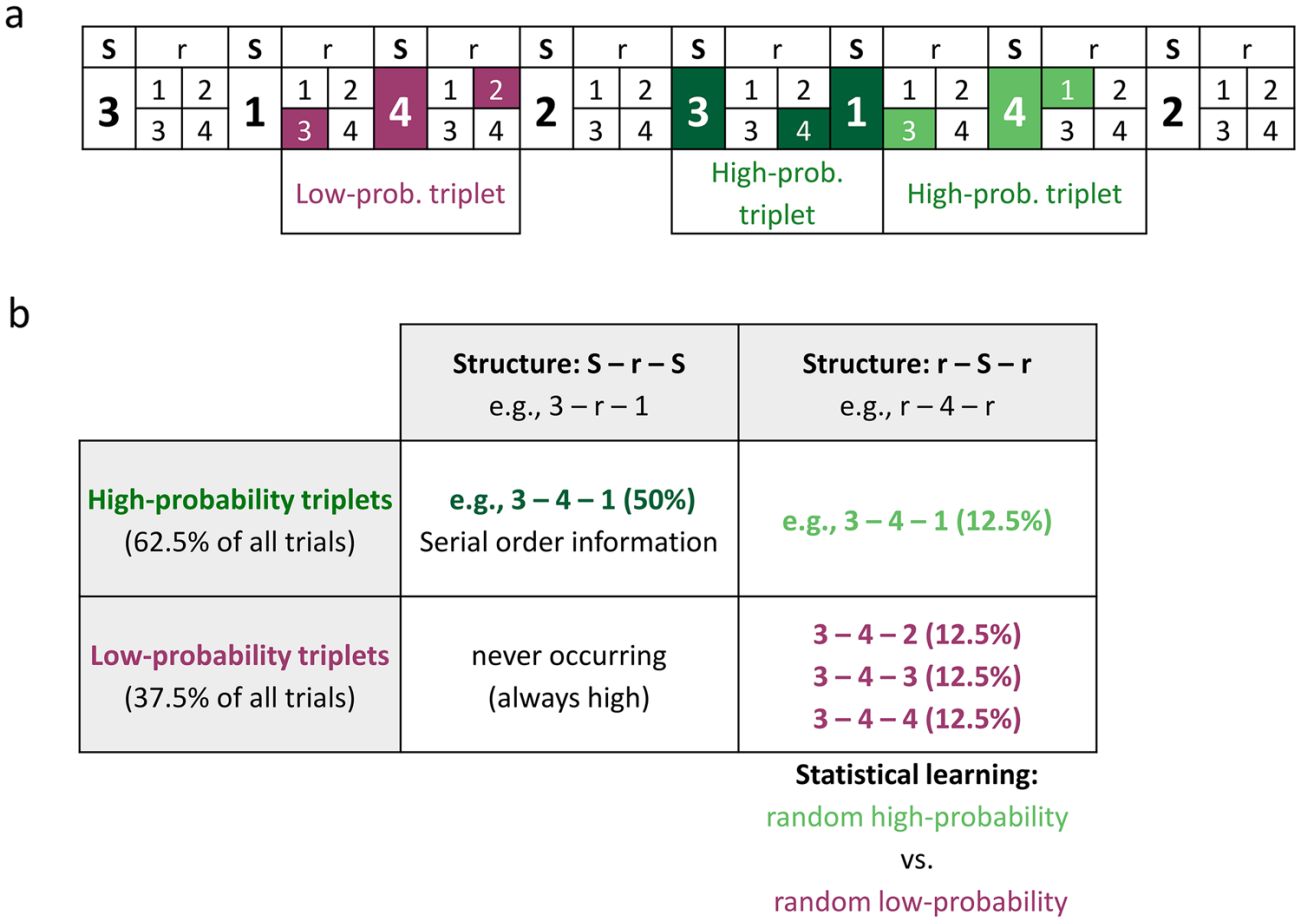
Stimulus structure and learning processes in the ASRT task (S—sequence, r—random). (**A**) As the ASRT task contains an alternating sequence structure (e.g., 2-r-3-r-1-r-4-r, where numbers correspond to the four locations on the screen and the ‘r’ represents randomly chosen locations out of the four possible ones), some associations of three consecutive trials (triplets) occur with greater probability (green) than others (purple). Within these triplets, due to the probabilistic structure, the third trial can be predicted by the first one with a certain probability, while the middle trial has no predictive value (i.e., second-order non-adjacent transitional probabilities). In the example above, the triplets 2-x-3, 3-x-1, 1-x-4, and 4-x-2 (where ‘x’ indicates the middle trial) are more probable. In contrast, e.g., 2-x-1, 1-x-3 or 3-x-2 would occur less probably. (**B**) We determined for each sequence and random trial whether it was the last trial of a high- or a low-probability triplet and, therefore, three different trials could occur: sequence (dark green, always high-probability), random high-probability (light green) and random low-probability (purple). Statistical learning is computed as the *difference* in responses to random high- vs. random low-probability trials. All sequence trials were excluded from the present analyses (for details see Methods).

**Figure 2:**
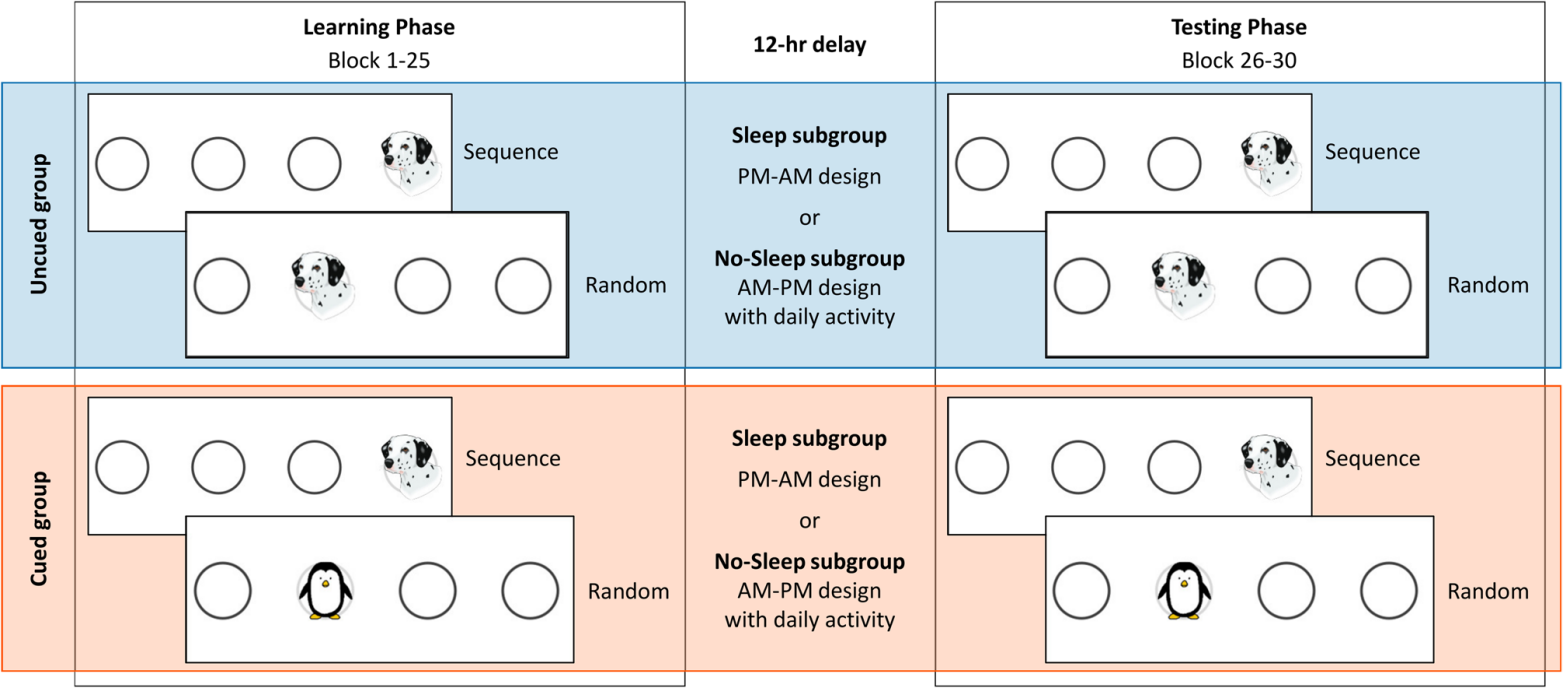
Design and procedure of the experiment. The ASRT task was administered in the experiment. In the cued version of the task (Cued group, orange panel), the regularity was marked by using different stimuli for the sequence trials (a dog’s head) and the random ones (penguin). In the uncued version of the task (Uncued group, blue panel), sequence and random trials were not marked differently (all stimuli were presented by the dog’s head). The Learning Phase (left column) consisted of 25 blocks, while the Testing Phase (right column) contained five blocks. The two sessions were separated by a 12-h delay. Based on the activity during the post-learning delay period, both main groups were divided into a Sleep (PM-AM design) and a No-sleep subgroup (AM-PM design). All participants performed the same ASRT task in the Testing Phase as in the Learning Phase.

One of the most prominent task paradigms to investigate statistical learning are probabilistic sequence learning tasks^12,25–30^. In the present study, we employed a widely used visuomotor probabilistic sequence learning task, the Alternating Serial Reaction Time (ASRT) task^25–27^ to assess visuomotor statistical learning. In this task, participants are asked to respond to a series of visually presented stimuli. The odd trials in the series follow a repeating serial order (a sequence), while the even trials are randomly selected, resulting in some runs of consecutive stimuli (second-order transitional probabilities) being more predictable than others. By cuing the sequence trials with different visual stimuli and instructing participants to learn their serial order while they *simultaneously* attend to and incidentally acquire the transitional probabilities in the same stimulus stream, the effect of divided attention on learning can be tested (see Figs. 1 and 2). That is, in this task design, cuing is assumed to divide attention between memorizing the repeating serial order of sequence trials and maintaining general task performance at the same time, while all stimuli remain attended. Although the effect of attention on statistical learning was not in the focus, so far, two studies investigated statistical learning in the cued version of the ASRT task comparing it to the uncued version. Whereas Nemeth et al.^26^ showed enhanced statistical learning by cuing the sequence, Szegedi-Hallgató et al.^31^ did not report such effect. Therefore, the question of whether sequence cuing, and thus divided attention, affects visuomotor statistical learning remains unclear.

Importantly, Nemeth et al.^26^ and Szegedi-Hallgató et al.^31^ used a self-paced task design, which enabled participants to spend as much time on the processing of and response to a given stimulus as they want. Such self-paced task designs can easily result in additional group differences and potential confounds as the acquisition process itself may become longer in the group completing the cued task version (spending more time on processing the cued stimuli) compared with the group that performs the task without cues. Therefore, in the present study, we modified the task design to better serve our aims. Specifically, we used a fixed time window, in which participants could process and respond to the stimuli, so that the maximum amount of time participants could spend on each trial was equal in the conditions with vs. without the attentional manipulation. Additionally, the employed fixed paced timing was relatively fast compared to the self-paced or other fixed paced versions of task^32–34^ to keep the attentional demand high in the Cued group. Due to these changes that were needed for the purpose of the present study, we refer to this task version as ‘cued’ instead of ‘explicit’ to differentiate it from the version used in previous studies.

As outlined above, previous studies have focused on how divided attention affects the acquisition of statistical knowledge, while how this knowledge is consolidated in the post-learning offline period that may contain sleep or daytime activities has been largely neglected. Typically—that is, without attentional manipulation—, statistical knowledge appears to be resistant to forgetting and interference, and the acquired knowledge is retained over an offline delay period^15,35–41^. Albeit a great body of research showed that sleep does not affect the consolidation of statistical knowledge, at least on the behavioral level^27,42–44^, other studies argued for a beneficial effect of sleep in this process^45,46^. So far, to the best of our knowledge, only one study tested the consolidation of statistical knowledge that was acquired under a cuing manipulation. They showed retained statistical knowledge tested after a relatively short (1.5-h) delay period, irrespective of the delay activity (napping, quiet rest or active rest^35^). Nevertheless, these results were not directly contrasted with performance in an uncued condition, and attention was not manipulated systematically. Our study contributes to this body of research by contrasting statistical knowledge that was acquired with vs. without attentional manipulation (cuing of the sequence) and testing the consolidation of this knowledge after a 12-h offline period of sleep vs. daytime wakefulness.

Overall, we aimed to investigate the effect of divided attention on the acquisition and consolidation of second-order transitional probabilities. Attention was manipulated by cuing: half of the participants received the cued version of the ASRT task (Cued group) and were instructed to discover and learn the order of the cued sequence trials, while the other half completed the uncued version (Uncued group) without any additional task (see Figs. 1 and 2). Importantly, we employed a fixed time window for stimulus presentation and response in both groups to control the maximum amount of time they spend on each trial. The fixed paced timing also helped ensure that the attentional demand remained high in the Cued group. To examine how the delay activity during the post-learning offline period affects the consolidation of statistical knowledge, we divided the two main groups (Cued and Uncued) into further subgroups: half of each (Cued/Uncued) group slept during the 12-h delay period (Sleep subgroups), and the other half had normal daily activities (No-sleep subgroups). In addition, to provide a fuller picture of the acquired statistical knowledge, the *Inclusion–Exclusion task*^47,48^ was administered: participants were asked to generate series of responses that followed the same regularities as the ASRT task (Inclusion condition) or one that was different from that (Exclusion condition). Based on Jacoby’s process dissociation procedure^49^, this task can help determine whether the acquired statistical knowledge remained implicit or participants could intentionally access and control this knowledge (for more details see the task description in the Methods section)*.*

The effectiveness of the cuing manipulation was assessed by the RTs on the repeating sequence trials as well as with sequence reports obtained during the short breaks in the ASRT task. The acquisition of second-order transitional probabilities (that is, statistical learning) was measured on the random trials, for which no specific learning instructions were given in either group. We expected that statistical learning is not affected by divided attention, as achieved by the cuing manipulation, and statistical knowledge is retained during the post-learning delay period, irrespective of cuing (cued vs. uncued) and the delay activity (sleep vs. wake). We also expected that statistical knowledge remains implicit in both groups.

## Methods

### Participants

Ninety-eight healthy young adults participated in the experiment. One participant was excluded due to technical errors and another participant due to outlier performance on the ASRT task [raw reaction times (RTs) fell outside three SDs consequently for the random trial types in both experimental sessions, for more details see the Statistical Analysis section as well]. Thus, the final sample consisted of 96 adults, 48 in each group. To measure the effect of delay activity on consolidation, both groups were divided into two subgroups (for more details, see the Procedure section): Twenty-four participants were assigned to the Uncued Sleep subgroup, 24 participants to the Uncued No-sleep subgroup, 23 participants to the Cued Sleep subgroup and 25 participants to the Cued No-sleep subgroup.

All participants had normal or corrected-to-normal vision, none of them reported a history of any neurological and/or psychiatric condition and drug-use. Prior to their inclusion in the study, participants provided informed consent to the procedure as approved by the research ethics committee of Eötvös Loránd University, Budapest, Hungary. The study was conducted in accordance with the Declaration of Helsinki and participants received course credits for taking part in the experiment.

Handedness was measured by the Edinburgh Handedness Inventory^50^. In this test, the laterality quotient (LQ) is assessed, which can vary between −100 and 100 where −100 indicates complete left-handedness and 100 indicates complete right-handedness. Dichotomic handedness (left or right) was defined based on the LQ (values above zero were coded as right-handedness, and values below zero as left-handedness). General cognitive performance was measured by three widely used neuropsychological tests: participants completed the Digit Span task^51,52^, the Counting Span task^53–56^, and the Attentional Network Test^57^. The four subgroups did not differ in either of these measures (Table 1, all *p*s > 0.088).

**Table 1:** Demographic data of the four subgroups.

Variable	Uncued group (N = 48)		Cued group (N = 48)	
	Sleep subgroup N = 24	No-sleep subgroup N = 24	Sleep subgroup N = 23	No-sleep subgroup N = 25
Age (year)	22.59 (4.52)	21.6 (2.57)	20.8 (1.15)	20.7 (1.62)
Education (year)	14.7 (2.43)	14.3 (2.18)	14.1 (1.09)	13.7 (2.16)
Handedness	19 right/4 left	19 right/5 left	19 right/4 left	19 right / 6 left
Gender	8 male/15 female	6 male/18 female	7 male/16 female	4 male / 21 female
Digit span task (short-term memory span, possible range: 3–9)	6.3 (1.26)	6.3 (0.87)	6.2 (0.97)	6.2 (0.93)
Counting span task (working memory span, possible range: 2–6)	3.5 (0.88)	3.6 (1.25)	3.9 (1.24)	3.3 (0.82)
ANT alerting attention (ms)	40.9 (23.0)	44.6 (27.96)	34.1 (18.80)	45.0 (29.45)
ANT orienting attention (ms)	31.4 (22.58)	26.4 (21.31)	38.8 (21.10)	41.1 (20.58)
ANT executive attention (ms)	88.4 (36.52)	77.5 (25.61)	84.83 (25.78)	89.1 (28.64)

Mean (SD) shown for age, education and standard neuropsychology tests assessing general cognitive performance. Ratio is presented for handedness and gender. *ANT*—Attention Network Test. Gender of one participant and handedness data from another participant are missing.

### Tasks

#### Alternating serial reaction time (ASRT) task

The ASRT task was used to measure the acquisition and consolidation of second-order transitional probabilities in the visuomotor domain. In this task, the target stimulus appeared in one of four horizontally arranged circles on the screen. Participants were instructed to respond with the corresponding key (Z, C, B or M on a QWERTY keyboard) when the stimulus occurred using their left and right middle and index finger. The presentation of the stimuli was determined by an eight-element sequence, within which sequence (S) and random (r) trials were alternating (Fig. 1A). One example of the sequence is 2-r-3-r-1-r-4-r, where numbers represent predetermined locations (1 denotes the leftmost position and 4 denotes the rightmost position) on the screen and r indicates randomly chosen locations out of the four possible ones. There are altogether six *unique* permutations of sequence order (1-r-2-r-3-r-4-r, 1-r-2-r-4-r-3-r, 1-r-3-r-4-r-2-r, 1-r-3-r-2-r-4-r, 1-r-4-r-3-r-2-r, 1-r-4-r-2-r-3-r). Please note that, for example, the sequences 1-r-2-r-4-r-3-r, 2-r-4-r-3-r-1-r, 4-r-3-r-1-r-2-r and 3-r-1-r-2-r-4-r consist of the same second-order transitional probabilities and are therefore treated as identical. Participants received one of the six unique sequences in a pseudo-random and counterbalanced manner.

The alternating structure resulted in a *probability* structure within which some chunks of three consecutive trials (*triplets*) occurred with greater probability than others (Fig. 1A). Triplets formed the second-order transitional probability information of the task: The last trial (N) of a triplet could be predicted by the first one (N-2) with a certain probability, while the interleaving trial (N-1) did not have a predictive value. For example, in the example sequence above, when 1 (N) appeared in a sequence position, it always followed 3 in the N-2 position, and the interleaving trial (N-1) was randomly selected. However, if 1 appeared in a random position, the N-2 trial could be any of the four trials by chance. Triplets were considered *high-probability* if the third trial was predicted by the first trial with a greater probability (62.5% probability, Fig. 1B), whereas triplets were considered *low-probability* if the third trial was predicted by the first trial with a lower probability (12.5% probability). Accordingly, participants could extract and acquire the highly probable associations. Importantly, triplets were identified as a moving window throughout the entire stimulus set, that is, each trial was categorized as the last element of either a high- or a low-probability triplet, and it was also the first or the second trial of the following triplets. There were 64 possible triplets in the task: 16 of them were high-probability triplets and 48 were low-probability ones.

Trials could also be defined based on their *sequence* property according to the sequence structure: the last trial of a triplet could occur in a sequence position (S-r-S structure; e.g., 3-x-1 in the example above) or in a random one (r-S-r structure; e.g., 2-x-1 in the example above). All triplets with S-r-S structure occurred with high-probability due to the repetition of the sequence and these took up 50% percent of all triplets (Fig. 1B). In contrast, triplets with the r-S-r structure could occur with either high- (12.5% of all triplets) or low-probability (37.5% of all triplets). Therefore, each trial had a probability and a sequence property, and the last trial of each triplet could be divided into the following three trial types: sequence (always high-probability), random-high and random-low trials. The repeating sequence trials were used to create the additional learning task in the Cued group: these trials could be visually differentiated from the random ones (i.e., they were cued), and participants were instructed to learn their serial order (for more details, see Procedure). Consequently, triplets with S-r-S structure were contaminated by the intentionally acquired sequence knowledge in the Cued group where the last element of these triplets could be predicted based on the sequence order rule instead of the probability-based rule. Therefore, we focused only on the random high- and random low-probability trials because the sole difference between these was probability-based, that is, purely statistical in nature^31^. *Statistical learning* (the acquisition of second-order transitional probabilities) was measured as the *difference* between the responses to random high- vs. random-low probability trials (Fig. 1B^26,31,33–35^). Previously, studies investigating this measure have shown that learning already occurs after a short period of practice and the acquired knowledge is well-preserved^26,31,36^. The underlying learning process can be tracked on the level of electrophysiological signals as well^33–35^.

Please note that previous studies mainly focused on the so-called triplet learning score calculated as the difference between the responses to high-probability versus low-probability trials without considering their sequence property. Thus, triplet learning score is not a pure measure of statistical learning because the probability information is contaminated by the serial order information. Despite this difference of whether or not sequence trials are included in the calculation of learning scores, the acquisition of second-order transitional probabilities (e.g., that ‘3-x-1′ is more probable than ‘2-x-1′ in the example sequence, Fig. 1) still contributes to both the triplet learning, and statistical learning scores. Importantly, statistical learning, triplet learning, and the acquisition of the sequence can be all separated from the so-called general skill learning that refers to general performance improvement as the task progresses (mainly due to improved visuomotor coordination) and affects the different trial types similarly^58^. In this study, we do not focus on these general changes in performance but only on statistical learning, that is, on the *difference* in responses to random high- vs. random low-probability trials.

#### Inclusion–exclusion task

The Inclusion–Exclusion task^47,48,59,60^ is based on the well-established ‘Process Dissociation Procedure’ (PDP^49^) and it is typically used to separate incidental and intentional use of memory. Accordingly, this task was administered to reveal whether or to what extent the acquired *probability-based associations* became intentionally available in both the Uncued and Cued groups. To this end, first, participants were asked to generate a series of responses that followed the same regularities (that is, including both sequence and random trials) as the ASRT task (Inclusion condition). Second, they were asked to generate a new series of responses that followed different regularities (again, including both sequence and random trials) than the learned one (Exclusion condition). Both conditions contained four runs and participants used the same response buttons as in the ASRT task. Each run finished after 24 button presses, which was equal to three rounds of the eight-element alternating sequence. To measure performance, we computed the percentage of high-probability triplets generated in the Inclusion and Exclusion conditions separately. We then tested whether participants produced more high-probability triplets than it would have been expected by chance (which was 25%, see Procedure), and whether the percentage of high-probability triplets differed across (Inclusion/Exclusion) conditions or across groups.

Following the standard analysis and interpretation of the task outlined in previous studies^34,36,61^, incidental use of knowledge (i.e., without intentional access to their knowledge) is sufficient to achieve good performance (that is, producing high-probability triplets *above* chance level) in the Inclusion condition. In contrast, good performance in the Exclusion condition (that is, producing high-probability triplets *at* or below chance level) requires intentionally accessible knowledge to exert control over their responses and generate a series of responses that is indeed different from what they practiced (i.e., intentionally exclude the acquired knowledge). Thus, if the acquired knowledge remained inaccessible to intentional control, we would expect high-probability triplets generated above chance level both in the Inclusion and Exclusion conditions. In contrast, intentional access to the acquired knowledge would result in generating high-probability triplets above chance level in the Inclusion condition and at or below chance level in the Exclusion condition.

### Procedure

The experiment consisted of two sessions to assess both learning and consolidation of the acquired knowledge. One block of the ASRT task contained 85 trials (stimuli). In each block, the eight-element alternating sequence repeated 10 times after five warm-up trials consisting only of random stimuli. The Learning Phase contained 25 blocks. Half of the participants received the uncued version of the ASRT task (*Uncued group*), while the other half performed a cued version of the task (*Cued group*) to induce divided attention. The Uncued group was informed that the aim of the experiment was to measure the effect of extended practice on motor performance, thus they were unaware of the sequence embedded in the task. In contrast, participants in the Cued group received information about the presence of a repeating sequence on the cued trials but not its length and were instructed to discover and intentionally memorize it as a secondary task. Nevertheless, participants were also instructed to pay attention to all stimuli and try to be equally fast and accurate on the pattern and random trials. In this version of the task, sequence and random stimuli were marked with different target pictures [dogs for sequence and penguins for random stimuli;^26,36^]. Consequently, the Uncued and Cued groups were named after the lack or presence of cues regarding the sequence. Please note that neither of the groups received any information regarding the second-order transitional probabilities of the task, therefore statistical learning is regarded as incidental in both groups.

To assess whether the learning situation remained incidental in the Uncued group, a short *questionnaire* was administered after the Testing Phase, similar to previous studies^27,62,63^. Participants were asked whether they observed any regularity in the task, and none of them reported anything regarding the embedded sequence or the learning situation. To ensure that the cuing manipulation was effective and the Cued group followed the instruction and learned the sequence order, a *post-block sequence report task* was administered after each block^33^. Participants were instructed to recall the order of the sequence trials (that was cued by the picture of a dog’s head) and report the order three times (12 button presses).

We used a fixed inter-stimulus interval (ISI) to control for the maximum amount of time that the Cued and Uncued groups can spend on a given trial and therefore on the whole task. The timing of an experimental trial was the following: The duration of stimulus presentation was 500 ms (when participants were required to respond to the stimulus), then the four empty circles were presented for 120 m before the next stimulus appeared, thus, the total ISI was 620 ms. These values are defined based on previous studies investigating healthy young adults, where participants had an average response time under 450 ms at the beginning of the task and 430 ms by the end of the Learning Phase^27,63–65^. Moreover, by using a fast-paced fix ISI, we also aimed to keep the attentional demand high in the Cued group throughout the task, as compared with a slow-paced ISI or self-paced timing where participants typically learn the repeating sequence already within the first three-six blocks^26,32,33,35,62^.

The Learning Phase was followed by a 12-h delay, thereafter the Testing Phase was administered, which contained five blocks of the ASRT task. In order to measure the effect of sleep on consolidation, a PM-AM vs. AM-PM design was used: during the delay, half of both Uncued and Cued groups slept (Sleep subgroups, PM-AM design) and the other half had normal daily activity (No-sleep subgroups, AM-PM design^27,66^). All PM sessions took place between 7 and 10 PM, and all AM sessions took place between 7 and 10 AM. Participants in the sleep subgroups slept on average 6 h (Uncued group: *M* = 5.8, *SD* = 0.84; Cued group: *M* = 6.0, *SD* = 0.84; *p* = 0.511). All participants were aware that they would perform the same task in the second experimental session (Fig. 2). Following the Testing Phase, both the Uncued and the Cued group performed the Inclusion–Exclusion Task.

### Statistical analysis

Statistical analyses were based on previous studies^25,26,33,35,36,67^ and were carried out using SPSS version 22.0 (SPSS, IBM).

#### Post-block sequence report task

This task was administered in the Cued group only. Performance on this task was used to test whether participants followed the instruction to learn the serial order of the cued sequence trials. Due to technical reasons, data from one participant in the Sleep subgroup was not recorded. First, the percentage of correct button presses (out of 12) was calculated after each block, then these were averaged across epochs (i.e., blocks of five, see below). These averaged values were submitted to mixed design ANOVAs separately for the Learning Phase and the 12-h post-learning offline delay to reveal whether participants followed the instruction and ascertain that the cuing manipulation was effective.

#### ASRT task

Epochs of five blocks were analyzed instead of single blocks: The Learning Phase consisted of five epochs, while the Testing Phase consisted of one epoch. On average, the Cued group showed slower RTs compared with the Uncued group, possibly as a result of the cuing manipulation (for more details see the Supplementary Materials). Hence, to ensure that potential between-group differences in statistical learning are not due to this group difference, we standardized the original RT values. To this end, first we transformed the data by dividing each participants’ raw RT values for the random trials with their own average performance of the first epoch of the Learning Phase [for a similar approach see^68^]. In the next step, we multiplied all data by 100 for easier interpretability and presentation. This way, results could be interpreted in terms of percentages, where each participants’ performance at the beginning of the Learning Phase was around 100% and changed as the task progressed. Following the transformation, the Uncued and Cued groups showed similar RTs (main effect of INSTRUCTION: *F*(1, 92) = 0.011, *p* = 0.916, η_*p*_^2^ < 0.001). Note that the results concerning raw RTs and accuracy can be found in the Supplementary Materials (Table S1 and Table S3, respectively, and Table S4 for the Bayesian analyses).

We calculated median RTs for correct responses only, for each participant and each epoch, separately for the random high- and random low-probability trial types. Then we calculated statistical learning scores as the *difference* between these two trial types, by extracting random high-probability trials from random low-probability ones. Larger scores indicated better learning performance. Due to the transformation procedure, these learning scores can be interpreted as percentages showing how much faster participants responded to the random high-probability trials compared with the random low-probability ones. These learning scores were submitted to mixed design ANOVAs to evaluate learning and consolidation of the acquired knowledge, respectively. Greenhouse–Geisser epsilon (ε) correction was used when necessary. Original *df* values and corrected *p* values (if applicable) are reported together with partial eta-squared (η_*p*_^2^) as the measure of effect size. LSD correction was used for pairwise comparisons and Cohen’s *d* is reported as an effect size.

As we expected no change in performance as a function of cuing and/or the delay activity either during the Learning Phase or over the post-learning offline period, we conducted Bayesian ANOVAs to overcome the limitations of the frequentist approach (i.e., null-hypothesis significance testing^69,70^) and gain statistical evidence for the possible null-results. Bayes Factors (BF_01_) were calculated using JASP (version 0.8.1.1, JASP Team, 2017). In these Bayesian ANOVAs, BF_01_ values reflect how well a model fits the data against the null model. The smaller the BF_01_ value is, the better the model predicts the data. BF_01_ value of the null model, which contains the grand mean only, is always 1 as it is compared to itself^71,72^. Additionally, we also present the evidence in the data for including a factor or an interaction of factors in a model quantified by BF_Exclusion_ values (inverse of BF_Inclusion_ values). BF_Exclusion_ values reflect the change from prior inclusion odds to posterior inclusion odds and can be interpreted in the same ways as BF_01_ values (i.e., the smaller the value, the stronger the evidence for including the given factor). Bayesian analyses conducted on the standardized RTs as well as on the Inclusion–Exclusion task are presented after the standard null-hypothesis testing effects in the Results section below. For the sake of completeness, we also present the Bayesian analyses conducted on the raw RTs and the accuracy data in the Supplementary Materials (Discussion section and Table S4).

#### Inclusion–exclusion task

Twelve participants (two in the Uncued No-sleep subgroup, two in the Uncued Sleep subgroup, five in the Cued No-Sleep subgroup, and three in the Cued No-sleep subgroup) were excluded from these analyses as they did not follow the instructions in either the Inclusion or the Exclusion condition (e.g., not using every response buttons). Data from one participant in the Uncued Sleep subgroup is missing due to technical problems. To contrast performance against chance level, we calculated the percentage of high-probability triplets participants generated and subtracted the value of chance level (25%, percentage of the 16 high-probability triplets out of the 64 possible triplets). These scores were then submitted to a mixed design ANOVA. Since we did not expect any difference across the groups and the sample size was reduced here, a Bayesian mixed design ANOVA was also conducted and reported similarly as described above.

## Results

### Did the cuing manipulation successfully induce divided attention in the Cued group?

To test whether the applied cuing manipulation was effective to induce divided attention in the Cued group, we conducted two different comparisons. First, we assessed responses for the *sequence trials*. Specifically, we compared performance on sequence trials with the average performance on random trials regardless of probability (i.e., averaged across random high- and random low-probability trials) across the four subgroups during the Learning Phase as well as over the 12-h post-learning offline period. Standardized RTs were analyzed here instead of raw RTs since general performance on the random trials was slowed down in the Cued group as reported above. Here we highlight the main result of interest only; all effects can be found in Table S2 of the Supplementary Materials. Importantly, in the Uncued group, we did not find any significant difference in RTs to sequence vs. random trials, whereas the Cued group showed faster responses to sequence trials compared with the random ones (Learning Phase: *p* = 0.001; over the 12-h delay: *p* = 0.002). This result demonstrates that the Cued group followed the cuing instruction and learned the repeating sequence trials, whereas this learning process was not apparent in the Uncued group. For more details, see also the Methods section and Figure S2 of the Supplementary Materials.

**Figure 3:**
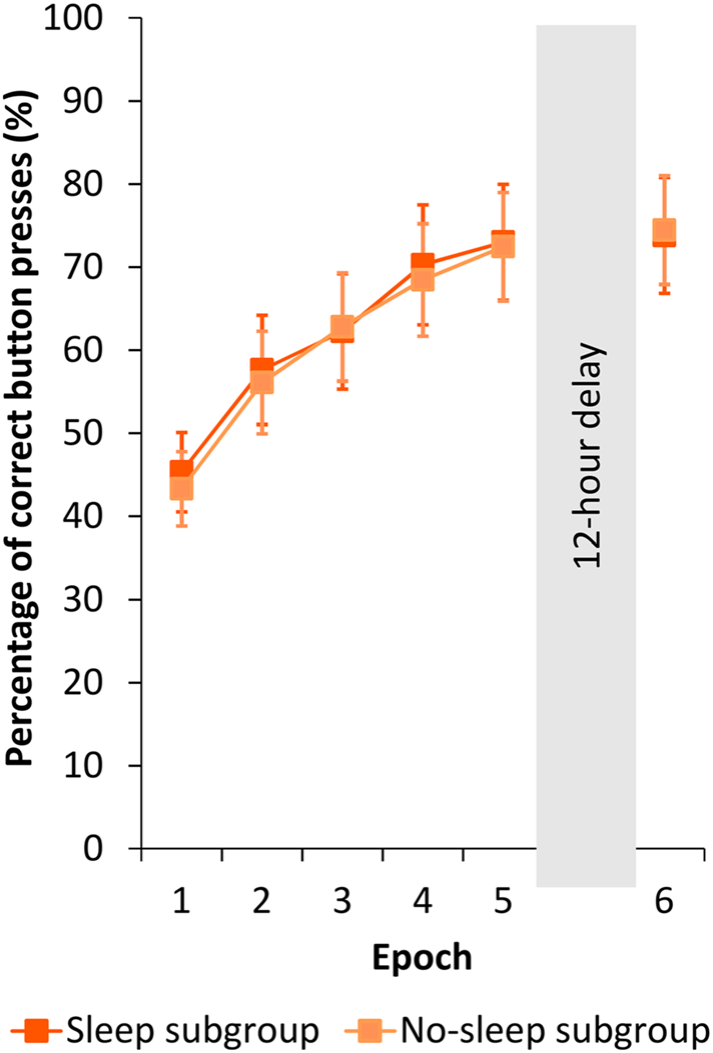
Performance of the Cued group in the post-block sequence report task over the Learning and Testing Phases. Participants were asked to provide 12 button presses, i.e., the four-element sequence three times, after each block of the ASRT task. Performance was defined as the percentage of correct button presses. The Sleep and No-sleep subgroups showed similar performance in the Learning Phase as well as after the post-learning offline period. While at the beginning of the Learning Phase participants performed around 40%, by the end of this session and during the Testing Phase they performed around 75% (i.e., they reported the sequence flawlessly two times out of three). We did not find any significant time of day or sleep-related effects during the Learning or Testing Phases, respectively. Error bars represent the standard error of the mean (SEM).

Second, we assessed the Cued group’s performance on the *post-block sequence report task* to ensure that they indeed acquired the sequence order. A mixed design ANOVA was conducted on the percentage of correct button-presses in the Learning Phase with EPOCH as a within-subject factor (1–5) and SLEEP (Sleep vs. No-sleep) as a between-subject factor. The analysis revealed significant sequence knowledge (INTERCEPT: *F*(1, 45) = 250.334, *p* < 0.001, η_*p*_^2^ = 0.848). Furthermore, knowledge of the sequence order gradually increased over practice (significant main effect of EPOCH: *F*(4, 180) = 19.078, *p* < 0.001, ε = 0.593, η_*p*_^2^ = 0.298), while performance was similar between the Sleep and the No-sleep subgroups (main effect of SLEEP: *F*(1, 45) = 0.02, *p* = 0.890, η_*p*_^2^ < 0.001; EPOCH × SLEEP interaction: *F*(4, 180) = 0.043, *p* = 0.974, ε = 0.593, η_*p*_^2^ = 0.001; Fig. 3). To assess performance over the 12-h delay period, a similar ANOVA was conducted with EPOCH (the last epoch of the Learning Phase and the first epoch of the Testing Phase; thus, Epoch 5 vs. Epoch 6) as a within-subject factor and SLEEP (Sleep vs. No-sleep) as a between-subject factor. Again, significant sequence order knowledge was found (INTERCEPT: *F*(1, 45) = 249.559, *p* < 0.001, η_*p*_^2^ = 0.847), while performance did not differ either as a function of epoch (main effect of EPOCH: *F*(1, 45) = 0.381, *p* = 0.540, η_*p*_^2^ = 0.008) or delay activity (main effect of SLEEP: *F*(1, 45) < 0.001, *p* < 0.995, η_*p*_^2^ < 0.001; EPOCH × SLEEP interaction: *F*(1, 45) = 0.068, *p* = 0.796, η_*p*_^2^ = 0.001; Fig. 3). Overall, the post-block sequence report task provided further evidence that participants followed the cuing manipulation.

### Do the uncued and cued groups show different statistical learning performance in the learning phase?

**Figure 4:**
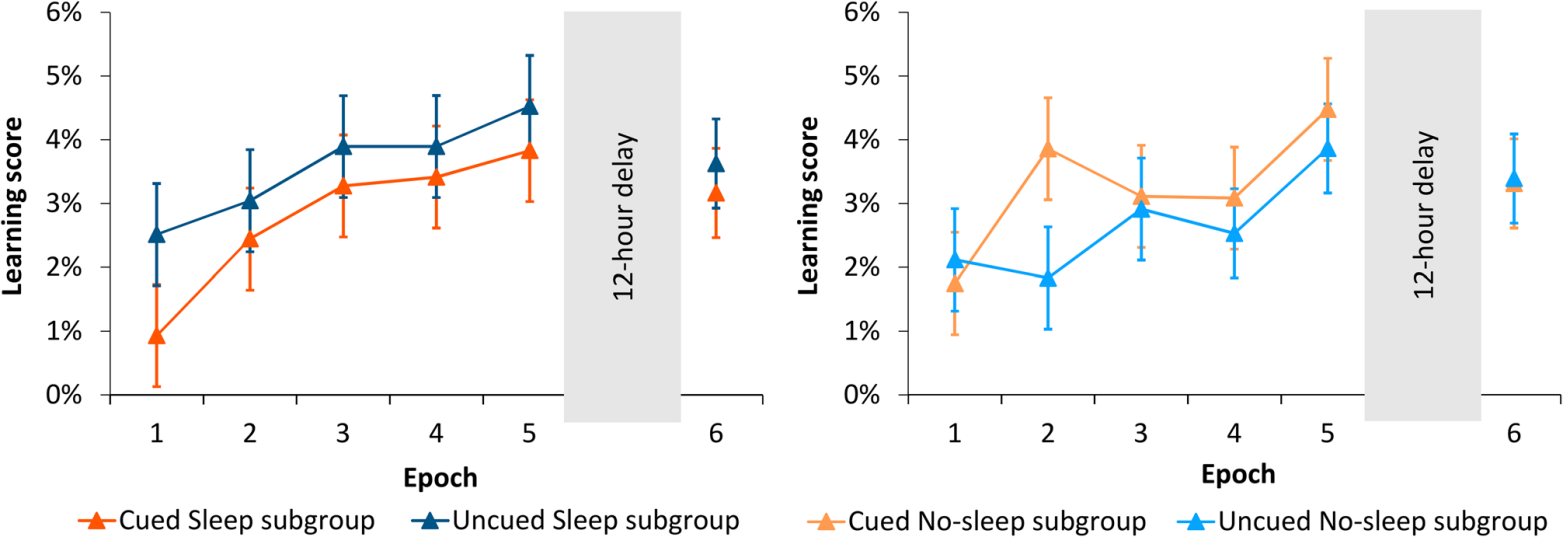
Statistical learning scores (i.e., the *difference* between RTs for random high- vs. random low-probability trials) over the Learning and Testing Phases. Left panel: Sleep subgroups within the Uncued and the Cued groups shown separately. Right panel: No-sleep subgroups within the Uncued and the Cued groups shown separately. The Uncued and Cued groups showed similar learning performance in the Learning Phase. Over the 12-h delay period, both the Uncued and Cued groups retained the acquired knowledge. We did not find any significant time of day or sleep-related effects during the Learning or Testing Phases, respectively. Error bars represent the SEM.

We tested potential group differences in statistical learning by conducting a mixed design ANOVA on standardized RT data of the statistical learning scores (i.e., *difference* between random high- vs. random low-probability trial types) with EPOCH (1–5) as a within-subject factor and INSTRUCTION (Uncued vs. Cued) and SLEEP (Sleep vs. No-sleep) as between-subject factors. Note that, for the Learning phase, the Sleep vs. No-sleep subgroup comparison can reflect time of day effects rather than the effect of sleep per se as the Sleep subgroup was first tested in the evening and the No-sleep subgroup was first tested in the morning. The ANOVA revealed significant statistical learning (INTERCEPT: *F*(1, 92) = 214.971, *p* < 0.001, η_*p*_^2^ = 0.700), that is, participants responded faster to random-high probability trials compared with the random-low probability ones. The Uncued and Cued group did not differ significantly in the degree of statistical learning (main effect of INSTRUCTION: *F*(1, 92) = 0.802, *p* = 0.374, η_*p*_^2^ = 0.011), indicating that the cuing manipulation did not affect this type of learning. Irrespective of the group instruction, statistical knowledge increased as the task progressed (significant main effect of EPOCH: *F*(4, 368) = 5.356, *p* < 0.001, η_*p*_^2^ = 0.055). The EPOCH × INSTRUCTION interaction was not significant (*F*(4, 368) = 0.671, *p* = 0.612, η_*p*_^2^ = 0.007), suggesting that the trajectory of statistical learning was also independent of the cuing manipulation (see Fig. 4).

The Sleep and No-sleep subgroups, irrespective of the cuing, showed similar overall degree of statistical learning (main effect of SLEEP: *F*(1, 92) = 0.288, *p* = 0.593, η_*p*_^2^ = 0.003) and did not differ in their learning trajectory (EPOCH × SLEEP interaction: *F*(4, 386) = 0.391, *p* = 0.815, η_*p*_^2^ = 0.004). The INSTRUCTION × SLEEP interaction neither reached significance (*F*(1, 92) = 2.800, *p* = 0.098, η_*p*_^2^ = 0.030), indicating that the time of day, irrespective of the cuing manipulation, did not affect statistical learning performance. The EPOCH x INSTRUCTION × SLEEP interaction was not significant either (*F*(4, 368) = 0.232, *p* = 0.920, η_*p*_^2^ = 0.003), indicating that the trajectory of statistical learning was comparable in the four subgroups.

To confirm our interpretations, a Bayesian mixed design ANOVA and BF_01_ values were calculated for the Learning Phase. Our data favored the model containing only the factor of EPOCH (BF_01_ = 0.0017; BF_Exclusion_ = 0.051), providing further support to a similar statistical learning process regardless of whether or not the sequence was cued as well as of the time of day when learning occurred. All other BF_01_ values were higher than 1 (INSTRUCTION: BF_01_ = 7.061, BF_Exclusion_ = 17.502; EPOCH × INSTRUCTION: BF_01_ = 3.860, BF_Exclusion_ = 103.581; SLEEP: BF_01_ = 6.507, BF_Exclusion_ = 16.079; EPOCH × SLEEP: BF_01_ = 5.640, BF_Exclusion_ = 159.104; INSTRUCTION × SLEEP: BF_01_ = 84.505, BF_Exclusion_ = 32.779; EPOCH × INSTRUCTION × SLEEP: BF_01_ = 479,224.597, BF_Exclusion_ = 187,945.864).

### Do the uncued and cued groups show different statistical performance over the 12-h offline period?

A similar mixed design ANOVA on statistical learning scores was conducted to assess consolidation after the offline period with EPOCH (the last epoch of the Learning Phase and the first epoch of the Testing Phase; thus, Epoch 5 vs. Epoch 6) as a within-subject factor and INSTRUCTION (Uncued vs. Cued) and SLEEP (Sleep vs. No-sleep) as between-subject factors. Overall, participants showed knowledge of the statistical regularities (significant INTERCEPT: *F*(1, 92) = 196.852, *p* < 0.001, η_*p*_^2^ = 0.681). Similarly to the Learning Phase, the Uncued and Cued groups did not differ significantly in the amount of statistical knowledge (main effect of INSTRUCTION: *F*(1, 92) = 0.083, *p* = 0.773, η_*p*_^2^ = 0.001; Fig. 4). Statistical knowledge was retained over the offline period (main effect of EPOCH: *F*(1, 92) = 2.704, *p* = 0.103, η_*p*_^2^ = 0.029), irrespective of the cuing manipulation (EPOCH × INSTRUCTION interaction: *F*(1, 92) = 0.055, *p* = 0.815, η_*p*_^2^ = 0.001).

There were no significant differences in overall learning scores between the Sleep and No-sleep subgroups (main effect of SLEEP: *F*(1, 92) = 0.002, *p* = 0.961, η_*p*_^2^ < 0.001), irrespective of the cuing manipulation (INSTRUCTION × SLEEP interaction: *F*(1, 92) = 0.620, *p* = 0.433, η_*p*_^2^ = 0.007). Both the Sleep and No-sleep subgroups retained the acquired knowledge over the delay period (EPOCH × SLEEP interaction: *F*(1, 92) = 0.002, *p* = 0.969, η_*p*_^2^ < 0.001), similarly in the Uncued and Cued groups (EPOCH × INSTRUCTION × SLEEP interaction: *F*(1,920) = 0.224, *p* = 0.637, η_*p*_^2^ = 0.002, Fig. 4).

A Bayesian mixed design ANOVA and BF_01_ values were calculated for the learning scores in Epoch 5 and 6. This analysis provided further support for our results: our data favored the null model (BF_01_ = 1), suggesting the retention of statistical knowledge over the offline period regardless of the delay activity (Sleep vs. No-sleep) and of the cuing manipulation (Uncued vs. Cued; see Fig. 4). All other BF_01_ values as well as BF_Exclusion_ values were higher than 1 (EPOCH: BF_01_ = 1.551, BF_Exclusion_ = 3.957; INSTRUCTION: BF_01_ = 5.109, BF_Exclusion_ = 12.780; EPOCH × INSTRUCTION: BF_01_ = 37.411, BF_Exclusion_ = 28.492; SLEEP: BF_01_ = 5.438, BF_Exclusion_ = 12.909; EPOCH × SLEEP: BF_01_ = 23.999, BF_Exclusion_ = 33.257; INSTRUCTION × SLEEP: BF_01_ = 88.567, BF_Exclusion_ = 48.228; EPOCH × INSTRUCTION × SLEEP: BF_01_ = 8951.745, BF_Exclusion_ = 1262.395).

### Was the acquired knowledge intentionally accessible?

**Figure 5:**
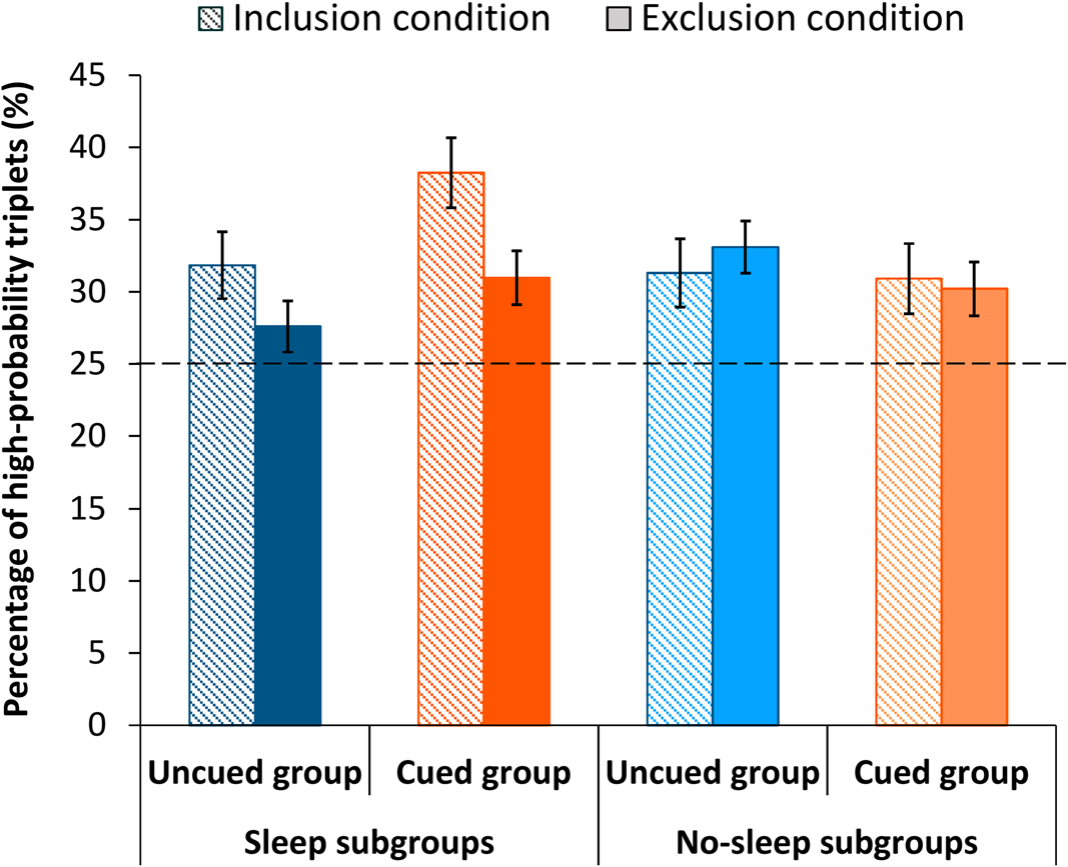
Percentage of high-probability triplets generated in the Inclusion and the Exclusion conditions by the four subgroups. Dashed horizontal line at 25% marks chance-level performance. Overall, participants performed above chance level both in the Inclusion and Exclusion conditions. The Sleep subgroups altogether (combined across the Uncued and Cued groups) generated a slightly different percentage of high-probability triplets in the Exclusion and Inclusion conditions, while the No-sleep subgroups performed at similar levels in the two conditions. This pattern seemed somewhat stronger for the Cued Sleep subgroup compared with the Uncued Sleep group. These group differences, however, were not supported by the Bayesian ANOVA. Error bars represent the SEM.

To test whether the acquired probability-based information remained implicit or became intentionally accessible, the *Inclusion–Exclusion task* was administered in both groups (see Methods) after the ASRT task of the Testing Phase. Data were analyzed in a mixed design ANOVA with CONDITION (Inclusion vs. Exclusion) as a within-subject factor and INSTRUCTION (Uncued vs. Cued) and SLEEP (Sleep vs. No-sleep) as between-subject factors. The significant main effect of INTERCEPT (*F*(1, 79) = 76.104, *p* < 0.001, η_*p*_^2^ = 0.491) revealed that, overall, participants generated high-probability triplets above chance level, which was 25%. The main effect of CONDITION revealed a slight trend towards more high-probability triplets being generated in the Inclusion than in the Exclusion condition (*F*(1, 79) = 3.205, *p* = 0.077, η_*p*_^2^ = 0.039). The main effect of INSTRUCTION (*F*(1, 79) = 0.254, *p* = 0.616, η_*p*_^2^ = 0.003) and the CONDITION × INSTRUCTION interaction (*F*(1, 79) = 0.901, *p* = 0.345, η_*p*_^2^ = 0.011) were not significant, suggesting that the cuing manipulation did not have an overall effect on the proportion of high-probability triplets generated in the Inclusion and Exclusion conditions (see Fig. 5), even despite the fact that the Cued group gained explicit knowledge about the order of the cued repeating sequence as evidenced by the results of the post-block sequence reports (see the first subsection of the Results).

The main effect of SLEEP was not significant (*F*(1, 79) = 0.254, *p* = 0.616, η_*p*_^2^ = 0.054). The CONDITION × SLEEP interaction, however, revealed a significant group difference between the Inclusion and Exclusion conditions (*F*(1, 79) = 4.687, *p* = 0.033, η_*p*_^2^ = 0.056). Pairwise comparisons showed that the Sleep subgroups altogether (i.e., combined across the Cued and Uncued groups) generated less high-probability triplets in the Exclusion condition compared with the Inclusion condition (*p* = 0.007, *d* = 0.09), while the No-sleep subgroups performed similarly in the two conditions (*p* = 0.791, *d* = 0.02). The INSTRUCTION × SLEEP interaction also revealed a significant effect (*F*(1, 79) = 4.419, *p* = 0.039, η_*p*_^2^ = 0.053). Pairwise comparisons on the *combined* performance in the Inclusion and Exclusion conditions showed that while the Sleep and No-sleep subgroups within the Uncued group performed comparably (*p* = 0.253, *d* = 0.05), the Cued Sleep subgroup generated slightly more high-probability triplets compared with the Cued No-sleep subgroup (*p* = 0.074, *d* = 0.10) and the Uncued Sleep subgroup (*p* = 0.030, *d* = 0.12), as well. The No-Sleep subgroups also performed similarly (*p* = 0.455, *d* = 0.03). This pattern seems to be driven mainly by the Cued Sleep subgroup generating more high-probability triplets in the Inclusion condition than any other subgroups (see Fig. 5), although the CONDITION × INSTRUCTION × SLEEP interaction did not reach significance (*F*(1, 79) = 0.009, *p* = 0.926, η_*p*_^2^ < 0.001). Overall, these results point towards that the Sleep subgroups could intentionally access and control the acquired knowledge to a certain level, while the No-sleep subgroups could not. This pattern seemed to be slightly stronger for the Cued Sleep subgroup compared with the Uncued Sleep subgroup. Nonetheless, as the effect sizes for the pairwise comparisons are small, these results should be treated with caution. Finally, all subgroups still performed above chance-level in the Exclusion condition, suggesting that none of them could exert full control over the acquired knowledge as required by the task instructions.

A Bayesian mixed design ANOVA was also conducted for this data, revealing that the best fitting model contains only the grand mean (BF_01_ of the null model = 1.000; CONDITION BF_01_ = 1.353, BF_Exclusion_ = 2.165; INSTRUCTION BF_01_ = 3.500, BF_Exclusion_ = 5.416; CONDITION × INSTRUCTION BF_01_ = 12.604, BF_Exclusion_ = 6.345; SLEEP BF_0_1 = 4.784, BF_Exclusion_ = 4.585; CONDITION × SLEEP BF_01_ = 1.353, BF_Exclusion_ = 1.838; INSTRUCTION × SLEEP BF_01_ = 12.454, BF_Exclusion_ = 3.735; CONDITION × INSTRUCTION × SLEEP BF_01_ = 28.641, BF_Exclusion_ = 5.918). Thus, the Bayesian analysis suggests that the delay activity related findings of the mixed-design ANOVA reported above are not reliable, possibly due to the low sample size, and therefore should be treated with caution.

## Discussion

The goal of the present study was twofold. First, we tested how divided attention affects the acquisition of second-order transitional probabilities in the same stimulus stream. Second, we investigated how the post-learning offline period affects the acquired statistical knowledge with a particular focus on the role of the delay activity (sleep vs. wake). Performance was measured by a probabilistic sequence learning (namely, the ASRT) task within which the sequence was cued for one half of the participants (Cued group) and uncued for the other half (Uncued group). We controlled for the time on task with a fast-paced fixed inter-stimulus interval, which also served to maintain the attentional demand in the cued version. The learning phase was followed by a 12-h post-learning offline period, which contained sleep for half of the groups (Sleep subgroups) and normal daily activity for the other halves (No-sleep subgroups), to measure consolidation of the acquired statistical knowledge. Compared with the Uncued group, the Cued group responded faster on the cued sequence trials than on the random ones, independent of trial-probability. Furthermore, the post-block sequence report task, administered in the Cued group only, showed that participants acquired the sequence order. These results provide evidence that participants in the Cued group followed the instruction and the cuing manipulation was successful in inducing a divided attention condition.

Our results regarding statistical learning performance revealed that the learning process was not sensitive to the cuing manipulation: The Cued and Uncued groups showed similar learning performance. The comparison of the Sleep and No-sleep subgroups in the Learning Phase could have reflected time of day related effects rather than the effect of sleep per se due to the applied PM-AM/AM-PM design. Nevertheless, we did not find such a time of day related group difference. After the 12-h post-learning offline period, all subgroups showed similarly retained statistical knowledge, suggesting that the consolidation was also independent of the cuing manipulation and was resistant to the delay, irrespective of the post-learning delay activity (sleep vs. wake). These results were further supported by the Bayesian analyses. Moreover, the analysis of raw (i.e., non-standardized) RTs as well as of the accuracy data also yielded the same patterns. The Inclusion–Exclusion task revealed that both the Cued and Uncued groups could comparably generate the knowledge on the structure of the task, while neither could intentionally access and control this knowledge. Sleep during the offline delay seemed to have a slightly beneficial effect on the latter process; nonetheless, this result should be treated with caution as the sample size was reduced here, and the Bayesian analysis did not support the finding.

In line with our hypothesis, the manipulation of attention had no effect on the *acquisition* of statistical knowledge, either as measured by RT learning scores (the primary measures for our analyses, see also Table S1 and Table S4 of Supplementary Materials) or by accuracy learning scores (see Table S3 and Table S4). So far, studies investigating statistical learning in the ASRT task with and without a cued sequence revealed mixed results. Nemeth et al.^26^ showed enhanced statistical learning performance, while in the study of Szegedi-Hallgató et al.^31^ performance was similar across the cued and uncued groups. The reason for these mixed results is unclear. One possible explanation is that the self-paced timing of the task enabled participants to spend different time on the task, potentially leading to enlarged individual differences within as well as across studies. Nevertheless, it is important to note that although these studies used a similar cued version of the ASRT task, they did not aim to systematically manipulate attention processes. We established changes in the experimental design so that it better suits the goals of the present study. First, we aimed to control for the time on task by using a fixed timing, so that the maximum amount of time that participants could spend on a given trial (and consequently on the task as a whole) was the same for the Uncued and Cued groups. Second, we applied a fast paced timing to avoid the automatization of sequence knowledge^32,33,35^ and keep the attentional demand high in the Cued group. Overall, our findings suggest that the acquisition of second-order transitional probabilities is not affected by divided attention in the ASRT task even when we control for the timing of the task.

Previous studies investigating the effect of a secondary task on statistical learning yielded inconsistent findings: Some conclude that statistical learning is resistant to a dual task manipulation^14,18–20,73^, while in other cases degraded performance is observed^16,19,21–24^. Importantly, statistical learning in the language domain seems to be more affected by a secondary task^16,22, cf. 23^, compared with statistical learning in the visuomotor domain^14,18–20^, in line with our results. However, as already mentioned in the Introduction, some of these studies used a secondary task related to a second stimulus stream resulting in a selective attention manipulation where good performance on both tasks can be achieved if attention is switched between the two tasks, potentially affecting the stimulus processing, as well^17,74,75^. In the present experiment, we chose the cuing of the repeating sequence embedded in the same stimulus stream as the probability-based associations, while all stimuli of that stream are similarly processed, and attention is divided between the cued and the uncued stimuli. Based on our and the previous results^14,18,20^, we conclude that visual statistical learning is not affected by divided attention.

Regarding the *consolidation* of statistical knowledge, on the one hand, we found that the acquired statistical information both with and without the cuing manipulation was comparably retained during the 12-h post-learning offline period. The consolidation of (pure) statistical knowledge has received relatively little empirical attention so far^15,46,76^. Previous studies that used the uncued version of the ASRT task only focused on the so-called triplet learning measure (for more details see Task section^36,37,41,42,66,77–79^), which, although somewhat contaminated with sequence information, seemed to be stable during the offline delay such as statistical learning. Consolidation of *pure* statistical knowledge in the cued version of the ASRT task has been investigated in Simor et al.’s study^35^, showing no change in statistical knowledge over a 1.5-h long offline delay. Our findings are consistent with these studies and our hypothesis, revealing reliably retained statistical knowledge during a 12-h offline period, irrespective of whether learning occurred with or without divided attention. These results were further confirmed by the Bayesian analysis, the raw (i.e., non-standardized) RT data and the accuracy data.

We also examined the role of delay activity (sleep vs. wake) in the consolidation of statistical knowledge, and found that sleep did not have a differential effect on performance, as expected based on previous studies^27,35,42,80,81^. The acquired knowledge was similarly retained, irrespective of the delay (sleep vs. wake) activity. Although the analysis of accuracies yielded improved consolidation during sleep as opposed to a wake offline period (see Results section and Table S3 of Supplementary Materials), this result was not confirmed by the Bayesian analysis (Discussion section and Table S3 in the Supplementary Materials). Furthermore, in line with our main results on standardized RTs, the analysis of raw RTs yielded evidence for a sleep-independent consolidation (for the frequentist analysis, see Introduction section, Figure S1 and Table S1; for the Bayesian analysis, see Discussion section and Table S4 in Supplementary Materials). Altogether, our results suggest that, at least in the visuomotor domain, statistical knowledge is retained over a 12-h delay period, irrespective of divided attention and delay activity (sleep vs. wake).

Finally, to investigate whether the cuing manipulation and the delay activity affected the intentional access to and control over the acquired statistical knowledge after the ASRT task in the Testing Phase, the *Inclusion–Exclusion task* adapted from Jacoby’s Process Dissociation Procedure^36,48,49^ was used. This measure revealed that the delay activity had a greater effect on performance than the cuing manipulation (cf. SLEEP × CONDITION interaction vs. INSTRUCTION × SLEEP interaction). The Sleep subgroups (combined across the Cued and Uncued groups) could intentionally access and control the acquired knowledge to a certain level, while the No-sleep subgroups could not. This pattern seemed somewhat stronger for the Cued Sleep subgroup compared with the Uncued Sleep as well as the Cued No-Sleep subgroup. Importantly, as the Bayesian analysis did not confirm these results and sample size was reduced in this task (see the Inclusion–Exclusion task subsection of Statistical analysis), these conclusions should be treated with caution. The lack of a significant INSTRUCTION × CONDITION interaction suggests that even though the Cued group intentionally learned the repeating sequence order (see the post-block sequence report task and the analysis of sequence trials), this did not improve the access to their statistical knowledge. Overall, results suggest that none of the groups could exert intentional control over the acquired probability-based knowledge.

The present study is not without limitations. First, opposing the established divided attention designs where the primary and secondary tasks can be tested both together and independently, the secondary task (i.e., the intentional acquisition of the sequence order) could not be tested separately in the present experiment. The main goal of the present study was to keep all stimuli attended and rule out that statistical learning performance is affected merely due to altered stimulus processing [cf. 17]. To this end, we chose an experimental design that, although did not allow us to measure the secondary task separately, bore the benefit that both tasks took place within the same stimulus stream. Second, the applied fixed paced timing could have resulted in biased RTs by disincentivizing participants to respond as fast as possible. Nevertheless, we still observed faster RTs for the sequence trials compared with the random ones in the Cued group (cf. Figure S2), suggesting that the task settings were still well-suited to assess the effect of cuing on performance. Third, the applied PM-AM/AM-PM design inevitably created group differences regarding the time of day when acquisition and testing took place. Although this difference could have confounded the present results, the lack of Sleep versus No-sleep subgroup differences in statistical learning or consolidation speaks against this scenario.

In summary, the present study showed that divided attention does not affect the acquisition and consolidation of second-order transitional probabilities in the visuomotor domain. Statistical learning successfully took place and the acquired knowledge was retained over the 12-h post-learning offline period, irrespective of whether or not participants paid attention to the cued sequence embedded in the same stimulus stream. Sleep seems to have no superiority compared with a wake delay activity in these processes. Overall, our findings provide deeper insights into the potential roles of divided attention and the post-learning delay activity (sleep vs. wake) in the acquisition and consolidation of statistical knowledge and highlight the robustness of these processes.

## Supplementary information

Supplementary Information 1.

## Data Availability

All data are available on the following online repository: https://osf.io/b68pg/?view_only=c22678446d0f47bfaa78d051f00e0af9.
