# Supplementary material for: Divided attention does not affect the acquisition and consolidation of transitional probabilities
Source: Sci Rep. 2020 Dec 31;10:22450. doi: 10.1038/s41598-020-79232-y (PMC7775459; doi:10.1038/s41598-020-79232-y)
Supplement: Supplementary file 1 — Supplementary Information 1. [file 41598_2020_79232_MOESM1_ESM.docx]

**Supplementary Materials for ‘Divided Attention does not affect the acquisition and consolidation of transitional probabilities’**

Kata Horváth, Csenge Török, Orsolya Pesthy, Dezso Nemeth, Karolina Janacsek

1. **Raw (non-standardized) RT performance on random high-probability vs. random-low probability trials**

Here we report performance on the random high-probability vs. random low-probability trials (i.e. statistical learning contrast) measured by raw RTs (Figure S1). A mixed-design ANOVA containing EPOCH (1-5 for the Learning Phase, 5-6 for the 12-hr delay) and TRIAL TYPE (random high-probability vs. random low-probability) as within-subject factors and INSTRUCTION (cued vs. uncued) and SLEEP (sleep vs. no-sleep) as between-subject factors was conducted for the Learning Phase as well as for the 12-hr post-learning offline delay. Below we highlight and summarize only those results that are relevant for the primary analyses and interpreted in the main text. For the detailed results see Table S1.

The Cued group showed significantly slower RTs on average compared with the Uncued group both in the Learning phase and after the 12-hour delay (significant main effect of INSTRUCTION; *p* < .001, *p* = .011, respectively). Slower RTs in the Cued group could suggest a higher attentional load, which could also indicate that the divided attention manipulation was effective, in line with the performance on sequence trials (see section 2, Table S2, and Figure S2 below) and on the post-block sequence report task (for more details see Results in the main text). The average RTs did not differ across the four subgroups when the SLEEP factor was also taken into account (the INSTRUCTION x SLEEP interaction not significant), however, the trajectory of average RTs differed across the four subgroups (significant EPOCH x INSTRUCTION x SLEEP interaction). Importantly, this latter effect did not involve the TRIAL TYPE factor, suggesting that the effect was independent of statistical learning, and therefore will not be discussed further. To control for group differences in average RTs in the subsequent analyses, raw RTs were standardized as presented in the main text (for more details see Statistical Analysis and Results). Nevertheless, it is important to note, that the TRIAL TYPE-related effects (that is, those related to statistical learning) in the raw RT ANOVAs are consistent with the results on standardized RTs presented in the main text as well as with the Bayesian analyses presented below in Table S4.

**
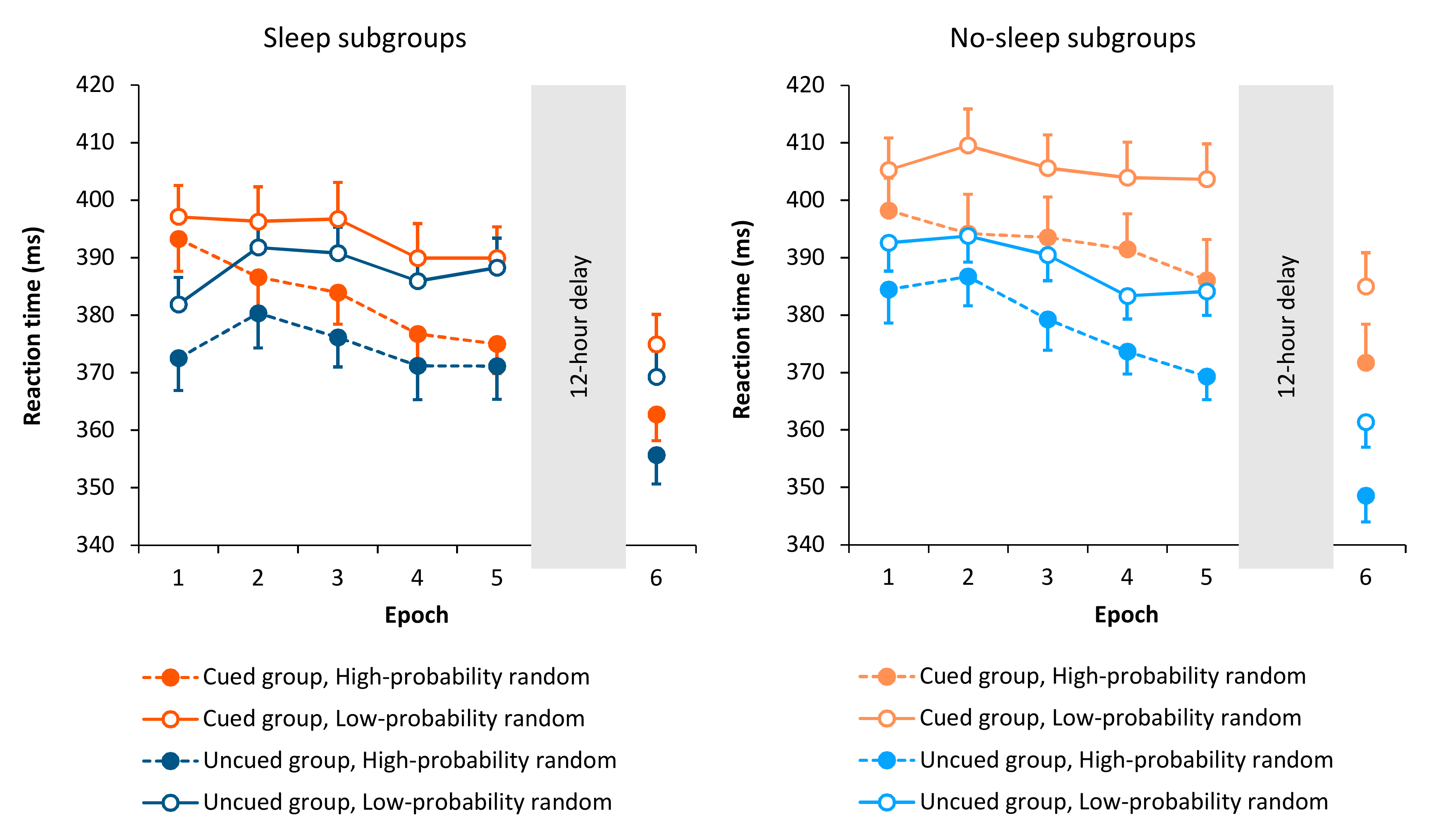
**

**Figure S1. Raw reaction times (RTs) for the random high-probability and random low-probability** **trials** over the time course of learning, separately for the four subgroups. The Cued group showed slower RTs on average compared with the Uncued group, while the difference between the random high- and low-probability trials, that is statistical learning, did not differ across groups. Error bars represent the standard error of the mean (SEM).

**Table S1.** ANOVA results for raw RTs on random high-probability vs. random low-probability trials.

|  | EPOCH | | | TRIAL TYPE | | | EPOCH * TRIAL TYPE | | | INSTRUCTION | | |
| --- | --- | --- | --- | --- | --- | --- | --- | --- | --- | --- | --- | --- |
|  | *F* | *p* | η*_p_*^2^ | *F* | *p* | η*_p_*^2^ | *F* | *p* | η*_p_*^2^ | *F* | *p* | η*_p_*^2^ |
| Learning Phase | 10.737 | **< .001** | .105 | 221.812 | **< .001** | .707 | 5.072 | **.001** | .052 | 5.518 | **.021** | .021 |
| 12-hr delay | 115.178 | **< .001** | .556 | 196.530 | **< .001** | .681 | 2.840 | .095 | .030 | 6.699 | **.011** | .068 |
|  | EPOCH x INSTRUCTION | | | TRIAL TYPE x INSTRUCTION | | | EPOCH x TRIAL TYPE x INSTRUCTION | | | SLEEP | | |
|  | *F* | *p* | η*_p_*^2^ | *F* | *p* | η*_p_*^2^ | *F* | *p* | η*_p_*^2^ | *F* | *p* | η*_p_*^2^ |
| Learning Phase | 1.206 | .307 | .013 | 0.002 | .963 | < .001 | 0.652 | .625 | .007 | 1.880 | .174 | .020 |
| 12-hr delay | 1.890 | .173 | .020 | 0.002 | .961 | < .001 | 0.037 | .848 | < .001 | 0.341 | .561 | .004 |
|  | EPOCH x SLEEP | | | TRIAL TYPE x SLEEP | | | EPOCH x TRIAL TYPE x SLEEP | | | INSTRUCTION x SLEEP | | |
|  | *F* | *p* | η*_p_*^2^ | *F* | *p* | η*_p_*^2^ | *F* | *p* | η*_p_*^2^ | *F* | *p* | η*_p_*^2^ |
| Learning Phase | 0.504 | .654 | .005 | 0.154 | .696 | .002 | 0.358 | .839 | .004 | 0.645 | .424 | .007 |
| 12-hr delay | 1.326 | .253 | .014 | 0.006 | .940 | < .001 | 0.001 | .991 | < .001 | 2.740 | .101 | .029 |
|  | EPOCH x INSTRUCTION x SLEEP | | | TRIAL TYPE x INSTRUCTION x SLEEP | | | EPOCH x TRIAL TYPE x INSTRUCTION x SLEEP | | |  | | |
|  | *F* | *p* | η*_p_*^2^ | *F* | *p* | η*_p_*^2^ | *F* | *p* | η*_p_*^2^ |  |  |  |
| Learning Phase | 2.842 | **.046** | .030 | 2.743 | .101 | .029 | .221 | .927 | .002 |  |  |  |
| 12-hr delay | 0.062 | .803 | .001 | 0.645 | .424 | .007 | 0.176 | .676 | .002 |  |  |  |

*Note*. *P* values < .05 are **bold-faced**. The main effect of EPOCH can indicate RT changes during the task, irrespective of trial type. The main effect of TRIAL TYPE can indicate RT differences between the random high- probability and random low-probability trial types (that is, statistical learning), while the EPOCH x TRIAL TYPE interaction can indicate different trajectories for these trial types in the time course of learning. The main effect of INSTRUCTION can indicate differences in average RTs between the Uncued and Cued groups, irrespective of trial type, while the EPOCH x INSTRUCTION interaction can indicate group differences in the time course of average RTs. The TRIAL TYPE x INSTRUCTION interaction can indicate trial type-related differences between the Uncued and Cued groups, and the EPOCH x TRIAL TYPE x INSTRUCTION interaction can indicate such differences in the time course of learning. The main effect of SLEEP can indicate differences in average RTs between the Sleep and No-sleep subgroups, irrespective of the cuing manipulation and trial type, while the EPOCH x SLEEP interaction can indicate group differences in the time course of average RTs. The TRIAL TYPE x SLEEP interaction can indicate trial type-related differences between the Sleep and No-sleep subgroups, irrespective of the cuing manipulation, and the EPOCH x TRIAL TYPE x SLEEP interaction can indicate such differences in the time course of learning. The INSTRUCTION x SLEEP interaction can indicate differences in average RTs across the four subgroups, irrespective of trial type, while the EPOCH x INSTRUCTION x SLEEP interaction can indicate differences in the time course of average RTs across the four subgroups. Finally, the TRIAL TYPE x INSTRUCTION x SLEEP interaction can indicate trial type-related differences across the four subgroups, while the EPOCH x TRIAL TYPE x INSTRUCTION x SLEEP interaction can indicate such differences in the time course of learning.

1. **Standardized RTs for sequence trials compared with random trials across the four subgroups**

To ensure that participants in the Cued group indeed followed the instruction regarding the sequence trials and, therefore, the divided attention manipulation was successful, we tested the performance on the sequence vs. random trials, irrespective of trial probability (that is, random trials included both high- and low-probability trials), across the four subgroups (see Figure S2). Here we report mixed design ANOVAs on standardized RTs with EPOCH (1-5 for the Learning Phase; 5 vs. 6 for the 12-hr delay) and TRIAL TYPE (Sequence vs. Random) as within-subject factors and INSTRUCTION (Uncued vs. Cued) and SLEEP (Sleep vs. No-sleep) as between-subject factors (for detailed results see Table S2). Importantly, these ANOVAs revealed a significant TRIAL TYPE x INSTRUCTION interaction both in the Learning and the Testing phases (*p* = .001, *p* = .002, respectively). The Cued group showed faster responses on the sequence trials compared with the random ones during the entire experiment (all *p*s < .001). In contrast, the Uncued group showed similar RTs on the sequence vs. random trials (Learning Phase: *p* = .913, 12-hr delay: *p* = .094). Overall, this result suggests that the Cued group followed the divided attention instruction throughout the task, and thus the manipulation was effective.

**
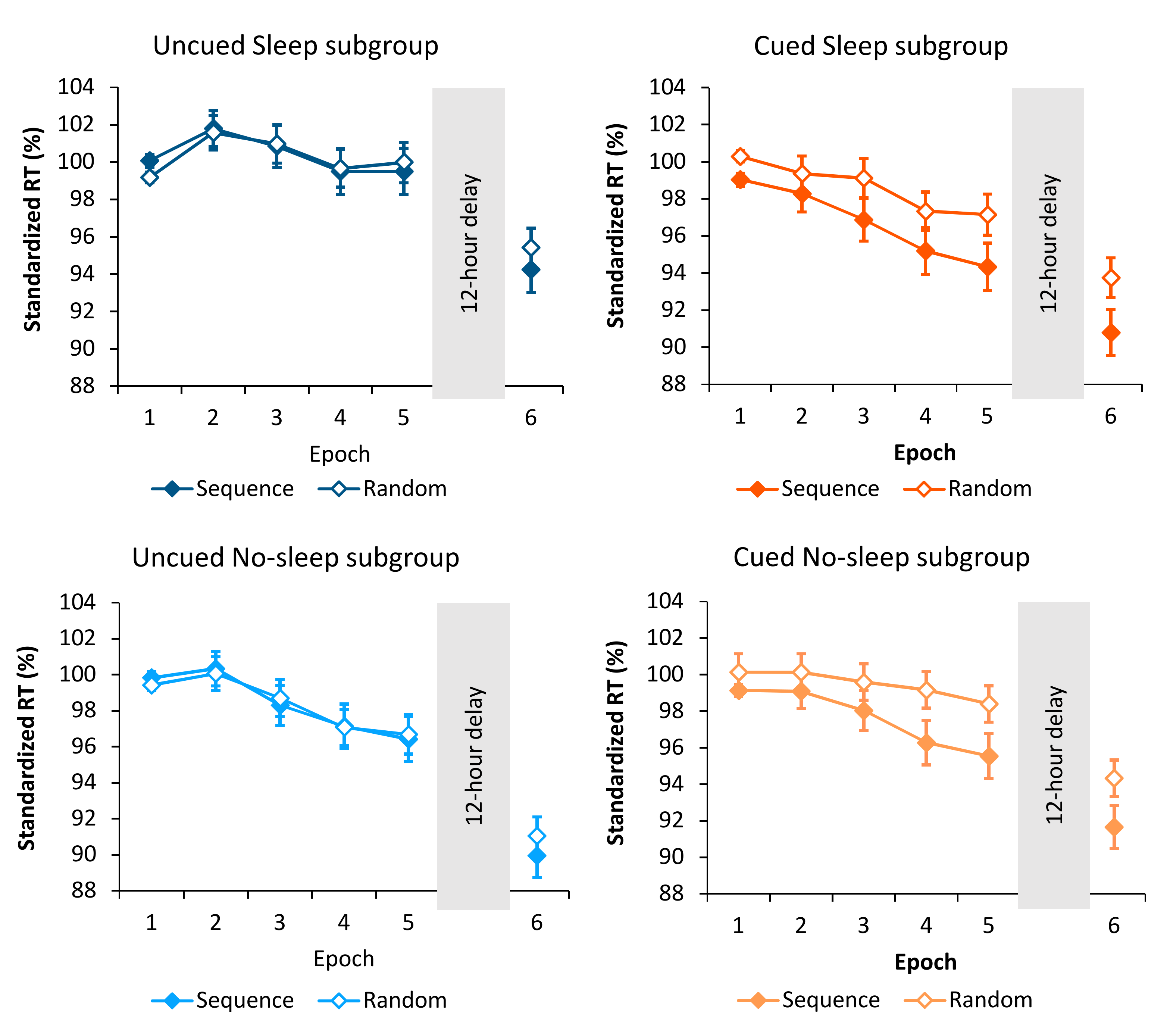
**

**Figure S2. Standardized RTs for the sequence and the random trials (irrespective of trial probability)** over the time course of learning across the four subgroups. While the Uncued Sleep and No-sleep subgroups showed similar RTs for trials in the sequence position as for trials in the random position, the Cued Sleep and No-sleep subgroups responded faster to the sequence trials compared with the random trials, indicating the effect of divided attention instruction. Error bars represent the SEM.

**Table S2.** ANOVA results for standardized RTs on sequence vs. random trials.

|  | EPOCH | | | TRIAL TYPE | | | EPOCH * TRIAL TYPE | | | INSTRUCTION | | |
| --- | --- | --- | --- | --- | --- | --- | --- | --- | --- | --- | --- | --- |
|  | *F* | *p* | η*_p_*^2^ | *F* | *p* | η*_p_*^2^ | *F* | *p* | η*_p_*^2^ | *F* | *p* | η*_p_*^2^ |
| Learning Phase | 18.531 | **< .001** | .169 | 11.700 | **.001** | .113 | 5.789 | **< .001** | .059 | 2.576 | .112 | .027 |
| 12-hr delay | 131.840 | **< .001** | .589 | 31.680 | **< .001** | .256 | 1.598 | .209 | .017 | .788 | .377 | .008 |
|  | EPOCH x INSTRUCTION | | | TRIAL TYPE x INSTRUCTION | | | EPOCH x TRIAL TYPE x INSTRUCTION | | | SLEEP | | |
|  | *F* | *p* | η*_p_*^2^ | *F* | *p* | η*_p_*^2^ | *F* | *p* | η*_p_*^2^ | *F* | *p* | η*_p_*^2^ |
| Learning Phase | 1.643 | .191 | .018 | 12.787 | **.001** | .122 | 1.121 | .344 | .012 | 0.482 | .489 | .005 |
| 12-hr delay | 4.808 | **.031** | .050 | 10.486 | **.002** | .102 | 1.748 | .189 | .019 | 1.845 | .178 | .020 |
|  | EPOCH x SLEEP | | | TRIAL TYPE x SLEEP | | | EPOCH x TRIAL TYPE x SLEEP | | | INSTRUCTION x SLEEP | | |
|  | *F* | *p* | η*_p_*^2^ | *F* | *p* | η*_p_*^2^ | *F* | *p* | η*_p_*^2^ | *F* | *p* | η*_p_*^2^ |
| Learning Phase | 0.438 | .677 | .005 | 0.001 | .993 | < .001 | 0.143 | .951 | .002 | 3.302 | .072 | .035 |
| 12-hr delay | 1.041 | .310 | .011 | 0.049 | .826 | .001 | 0.024 | .877 | < .001 | 5.358 | .023 | .055 |
|  | EPOCH x INSTRUCTION x SLEEP | | | TRIAL TYPE x INSTRUCTION x SLEEP | | | EPOCH x TRIAL TYPE x INSTRUCTION x SLEEP | | |  | | |
|  | *F* | *p* | η*_p_*^2^ | *F* | *p* | η*_p_*^2^ | *F* | *p* | η*_p_*^2^ |  |  |  |
| Learning Phase | 2.353 | .089 | .025 | 0.006 | .939 | .006 | 0.664 | .596 | .007 |  |  |  |
| 12-hr delay | 0.154 | .695 | .002 | 0.001 | .979 | < .001 | 0.167 | .683 | .002 |  |  |  |

*Note*. *P* values < .05 are **bold-faced**. For how to interpret these effects, please see the note of Table S1, with the only difference that the ANOVAs reported here contrasted sequence vs. random trials, instead of random high- vs. random-low probability trials. The ANOVAs revealed some differences in average RTs and their trajectories across groups (INSTRUCTION x SLEEP, EPOCH x INSTRUCTION x SLEEP, and EPOCH x INSTRUCTION interactions). Importantly, none of these effects involved the TRIAL TYPE factor and, therefore, it will not be further discussed. The interested reader could gain further insights into these effects on average RTs by inspecting Figure S2.

1. **Acquisition and consolidation of statistical knowledge measured by accuracy**

Similar ANOVAs were conducted for the statistical learning scores calculated on accuracy data as the ones reported in the main text using standardized RT data (see Results). The details of these ANOVAs can be found in Table S3. While most of the effects are consistent with the results on the standardized as well as raw RT data, the main effect of SLEEP and the EPOCH x SLEEP interaction during the 12-hr delay revealed an additional trend (*p* = .079; *p* = .061, respectively). Learning scores in the Testing Phase appeared to be slightly decreased when the offline delay contained wake activity (*p* = .023; end of the Learning Phase: 3.6%; Testing Phase: 1.6%) compared with sleep (*p* = .693; end of the Learning Phase: 3.7%; Testing Phase: 4.1%). This additional trend suggests a beneficial effect of sleep on consolidation regardless of attention manipulation, contrary to the standardized and raw RT effects (see the Results section in the main text and Table S4 below, respectively). This difference might be originating from accuracy and RT measures capturing different aspects of learning/memory: It has been previously argued that while RTs could reflect involuntary processes, accuracy could reflect voluntary and more controlled processes [1]. It is important to note, however, that this accuracy effect should be treated with caution as it was not supported by the Bayesian analysis (see Table S4). Moreover, previous studies reporting accuracy data found retention of the acquired knowledge [2–5], irrespective of post-learning delay activity [2,4]. Therefore, further studies are needed to confirm or disprove this effect.

**Table S3.** ANOVA results for statistical learning scores calculated on the accuracy data.

|  | INTERCEPT | | | | EPOCH | | | | INSTRUCTION | | | EPOCH x INSTRUCTION | | | |  |
| --- | --- | --- | --- | --- | --- | --- | --- | --- | --- | --- | --- | --- | --- | --- | --- | --- |
|  | *F* | *p* | η*_p_*^2^ | *F* | | *p* | η*_p_*^2^ | *F* | | *p* | η*_p_*^2^ | | *F* | *p* | η*_p_*^2^ | |
| Learning Phase | 88.848 | **< .001** | .491 | 5.506 | | **< .001** | .056 | .802 | | .373 | .009 | | 1.336 | .256 | .014 | |
| 12-hr delay | 80.127 | **< .001** | .466 | 1.772 | | .186 | .019 | 1.142 | | .288 | .012 | | 0.119 | .731 | .001 | |
|  | SLEEP | | | EPOCH x SLEEP | | | | INSTRUCTION x SLEEP | | | | | EPOCH x INSTRUCTION x SLEEP | | | |
|  | *F* | *p* | η*_p_*^2^ | *F* | | *p* | η*_p_*^2^ | *F* | | *p* | η*_p_*^2^ | | *F* | *p* | η*_p_*^2^ | |
| Learning Phase | 0.415 | .521 | .005 | 1.497 | | .202 | .016 | 0.011 | | .918 | < .001 | | 1.248 | .290 | .013 | |
| 12-hr delay | 3.150 | .079 | .033 | 3.602 | | .061 | .038 | 0.003 | | .956 | < .001 | | 0.295 | .588 | .003 | |

*Note*. *P* values < .05 are **bold-faced**. Since the ANOVAs are conducted on learning scores, the INTERCEPT can indicate significant learning. The main effect of INSTRUCTION can indicate INSTRUCTION differences in the learning scores. The main effect of EPOCH can indicate changes in the learning scores during the task, while the INSTRUCTION x EPOCH interaction can indicate INSTRUCTION differences in the time course of learning. The main effect of SLEEP can indicate Sleep and No-sleep SLEEP differences irrespective to the level of intention to learn. The INSTRUCTION x SLEEP interaction can indicate the SLEEP differences within the main INSTRUCTIONs, while the INSTRUCTION x EPOCH x SLEEP interaction can indicate these differences in the time course of learning. For more details, see the main text of the manuscript.

1. **Bayesian ANOVAs conducted on the raw RT and accuracy data**

Finally, here we present all BF_01_ and BF_Exclusion_ values for the Bayesian ANOVAs conducted on the raw RT and accuracy data (see Table S4). Please note that although raw RTs are presented separately for the random high-probability and random low-probability trials above (Figure S1, Table S1), here we report the Bayesian ANOVA conducted on learning scores for better comparability with the analysis presented in the main text as well as with the analysis conducted on accuracy data.

These analyses are in line with and support most results of the frequentist analyses (i.e., null-hypothesis significance testing). Notably, the slight trend for the effect of sleep on consolidation found in the accuracy data (see the previous section above) is not confirmed by the Bayesian ANOVA, supporting the conclusion that this effect should be treated with caution.

**Table S4.** Bayesian ANOVA results for statistical learning scores calculated on the raw RT and accuracy data.

| Model | Raw RTs | | | | Accuracy | | | |
| --- | --- | --- | --- | --- | --- | --- | --- | --- |
|  | Learning Phase | | 12-hr delay | | Learning Phase | | 12-hr delay | |
|  | BF_01_ | BF_Exclusion_ | BF_01_ | BF_Exclusion_ | BF_01_ | BF_Exclusion_ | BF_01_ | BF_Exclusion_ |
| NULL MODEL | 1.000 | **-** | **1.000** | - | 1.000 | **-** | **1.000** | **-** |
| EPOCH | **0.029** | **0.081** | 1.478 | 3.278 | **0.002** | **4.629e^-4^** | 2.515 | 4.255 |
| INSTRUCTION | 7.676 | 19.246 | 5.320 | 13.109 | 3.412 | 6.802 | 3.437 | 7.752 |
| EPOCH x INSTRUCTION | 6.567 | 113.944 | 34.553 | 30.268 | 0.018 | 5.747 | 35.247 | 20.408 |
| SLEEP | 7.278 | 17.832 | 5.289 | 12.947 | 5.504 | 12.500 | 1.460 | 2.740 |
| EPOCH x SLEEP | 10.139 | 175.568 | 35.192 | 32.186 | 0.074 | 20.408 | 2.945 | 2.994 |
| INSTRUCTION x SLEEP | 102.293 | 52.811 | 82.730 | 48.507 | 89.200 | 41.667 | 18.341 | 14.085 |
| EPOCH x INSTRUCTION x SLEEP | 94290.995 | 2262483.216 | 9499.680 | 1037.228 | 13.192 | 1000.000 | 512.674 | 11.111 |

*Note*. The BF_01_ value of the best fitting model is **bold-faced**. In Bayesian ANOVAs, BF_01_ values reflect how well a model fits the data: The smaller the BF_01_ value is, the better the model predicts the data. BF_01_ value of the null model, which contains the grand mean only is always 1 [6]. The BF_Exclusion_ value quantifies the evidence for the inclusion of a factor or an interaction of factors in the model and can be interpreted in the same direction as BF_01_ values (i.e. the smaller the value, the stronger the evidence for including the given factor). For how to interpret the main effects and interactions see the note of Table S3.

**References**

[1] Janacsek, K., Fiser, J. & Nemeth, D. The best time to acquire new skills: Age‐related differences in implicit sequence learning across the human lifespan. *Dev. Sci.* **15**, 496–505 (2012).

[2] Simor, P. *et al.* Delta and theta activity during slow-wave sleep are associated with declarative but not with non-declarative learning in children with sleep-disordered breathing. (2017) doi:10.1556/2053.01.2017.003.

[3] Kóbor, A., Janacsek, K., Takács, Á. & Nemeth, D. Statistical learning leads to persistent memory: Evidence for one-year consolidation. *Sci. Rep.* **7**, 760 (2017).

[4] Song, S., Howard, J. & Howard, D. Sleep does not benefit probabilistic motor sequence learning. *J. Neurosci.* **27**, 12475–12483 (2007).

[5] Romano, J. C., Howard Jr, J. H. & Howard, D. V. One-year retention of general and sequence-specific skills in a probabilistic, serial reaction time task. *Memory* **18**, 427–441 (2010).

[6] Jarosz, A. F. & Wiley, J. What are the odds? A practical guide to computing and reporting Bayes factors. *J. Probl. Solving* **7**, 2 (2014).
